# Helminth Parasites and the Modulation of Joint Inflammation

**DOI:** 10.1155/2011/942616

**Published:** 2011-04-18

**Authors:** Chelsea E. Matisz, Jason J. McDougall, Keith A. Sharkey, Derek M. McKay

**Affiliations:** ^1^Gastrointestinal Research Group, Department of Physiology and Pharmacology, The Calvin, Phoebe and Joan Snyder Institute of Infection Immunity and Inflammation, 1877 HSC University of Calgary, 3330 Hospital Drive NW, Calgary, AB, Canada T2N 4N1; ^2^Inflammation Research Network, Department of Physiology and Pharmacology, The Calvin, Phoebe and Joan Snyder Institute of Infection Immunity and Inflammation, University of Calgary, 1877 HSC, 3330 Hospital Drive NW, Calgary, AB, Canada T2N 4N1; ^3^Hotchkiss Brain Institute, University of Calgary, Calgary, AB, Canada T2N 4N1

## Abstract

There is an urgent need to develop better therapeutics for autoimmune and autoinflammatory diseases, of which musculoskeletal disorders such as rheumatoid arthritis are particularly prevalent and debilitating. Helminth parasites are accomplished masters at modifying their hosts' immune activity, and so attention has focused on rodent-helminth model systems to uncover the workings of the mammalian immune response to metazoan parasites, with the hope of revealing molecules and/or mechanisms that can be translated into better treatments for human autoimmune and idiopathic disorders. Substantial proof-of-principal data supporting the concept that infection with helminth parasites can reduce the severity of concomitant disease has been amassed from models of mucosal inflammation. Indeed, infection with helminth parasites has been tried as a therapy in inflammatory bowel disease, and there are case reports relating to other conditions (e.g., autism); however, the impact of infection with parasitic helminths on musculoskeletal diseases has not been extensively studied. Here, we present the view that such a strategy should be applied to the amelioration of joint inflammation and review the literature that supports this contention.

## 1. Introduction

Infection with helminth parasites results in a conserved series of immune events that are orchestrated and dominated by T helper cell type 2 (Th2) events [1]. Given the reciprocity in immune regulation where, for example, Th2 cell-derived mediators inhibit the activity of Th1 cells, the hypothesis arises that individuals infected with helminth parasites could be less susceptible to other inflammatory diseases. By extrapolation, infection with helminth parasites could be used to treat disease driven by Th1 cells. Many species of parasitic helminths reside at mucosal surfaces, and it has repeatedly been shown that infection with trematode, cestode, or nematode parasites can reduce the severity of colitis and airway inflammation in murine models [2]. The impact of infection with helminths on organs outside the parasite's location has received less attention. Here, we review the effect of infection with helminth parasites on joint inflammation.

The last four decades have seen an alarming increase in the prevalence of autoimmune diseases such as inflammatory bowel disease (IBD), diabetes, multiple sclerosis, and rheumatoid arthritis (RA) in Western societies. Rheumatoid arthritis is a painful and debilitating disease that affects ~1% of the North American population [3]. The socioeconomic burden of this disease is substantial; in 2003 arthritis and other rheumatic conditions cost the United States $127.8 billion per year, an amount equivalent to 1.2% of the GDP and tantamount to a small, chronic recession [4].

Current therapies for RA include nonsteroidal anti-inflammatory drugs (NSAIDs), glucocorticoids (GCs), and disease-modifying antirheumatic drugs (DMARDs). As their name implies NSAIDs inhibit inflammation, although their mild-to-moderate effects do not fully alleviate the symptoms of RA. Moreover, these drugs do not inhibit the progression of arthritic disease; thus joint damage persists. Adverse reactions in the gastrointestinal tract (GIT), kidney, and central nervous systems are frequently reported with the use of NSAIDs [5]. Glucocorticoids possess potent immunosuppressive and anti-inflammatory properties [6], but their use comes with considerable long-term risks: for example, GCs decrease calcium absorption and impair bone formation, leading to a significant decline in bone mineral density within the first 6–12 months of therapy [6]. Additional side effects of sustained GC use include weight gain, redistribution of adipose tissue, and increased risk of developing diabetes mellitus, peptic ulcers, pancreatitis, cataracts, and glaucoma [6]. While DMARDs have been successful in slowing the progression of bone and cartilage erosion, these drugs (e.g., methotrexate (MTX)), even at low doses, can generate a variety of toxic side effects [7]. Toxic effects of MTX have been observed in the liver and GIT, mucous and skin membranes, and the respiratory and hematopoietic systems [7].

Despite many pharmacological advances, the limited effectiveness and adverse side effects of current therapies for arthritic diseases highlight the urgent need for alternative treatment(s), which can only be generated through a better understanding of the mediators and mechanisms that generate pain and inflammation in the joint [8]. While animal models do not fully replicate human RA, they are invaluable tools for elucidating immunopathological processes that can cause arthritis. For example, the inhibition of arthritic disease in mice by neutralizing the proinflammatory cytokine, tumor necrosis factor (TNF)-*α*, has led to the introduction of anti-TNF*α* therapy in clinical practice [9].

## 2. Animal Models of Joint Inflammation

Numerous studies suggest that RA is more accurately classified as a syndrome rather than one specific disease, as evidenced by the complex pathophysiological pathways involved. There exists a wide diversity of models of joint inflammation that employ rabbits, guinea pigs, and primates, although rodents are, by far, the most commonly used (Table 1). The induction of arthritis in these models typically employs the administration of an adjuvant ± an antigen, with the antigen immunization generating immunological memory. The use of transgenic mouse technology and the development of knock-out strains of mice have provided the opportunity to examine the “spontaneous” development of arthritis, and the genes and proteins that contribute to joint inflammation.

The most commonly used model of arthritis is the type II collagen (Col II) model. Collagen is a key component of cartilage, and immunologic hypersensitivity to Col II results in joint inflammation. In this model, animals are immunized with type II collagen (from the same or a different species) that is emulsified in Freund's adjuvant. This provokes a multijoint inflammation characterized by synovitis, inflammatory cell infiltrate, pannus formation, bone and cartilage erosion, and fibrosis: features that more closely resemble human RA than those observed with adjuvant only-induced arthritis [26]. The source of collagen used for immunization affects the character of the disease: heterologous (from a different species) Col II elicits an acute and severely erosive polyarthritis 2-3 weeks after immunization, while homologous Col II evokes a severe and more chronic form of arthritis [24].

Arthritis is commonly induced through the use of Freund's adjuvant (mineral oil) with (Freund's Complete adjuvant; CFA) or without (Freund's incomplete adjuvant; IFA) the addition of an inactivated bacterium (frequently *Mycobacterium tuberculosis*). CFA is effective in stimulating cell-mediated immunity, leading to the production of antibodies. Depending on the route of administration, a systemic or local arthritic effect can be induced. Intra-articular injection of CFA results in a monoarthritis which spares the other joints, and while not fully recapitulating RA this offers the advantage of a localised inflammation with reduced systemic involvement and allows for pain assessment studies in which the contralateral, noninjected joint acts as an internal control. The CFA model is often used for testing potential anti-inflammatory or antinociceptive therapeutic agents.

The most recent addition to the panoply of models of arthritis has been the use of transgenic animals engineered to either overexpress or to lack a particular gene either globally or in a specific tissue. For example, overexpression of TNF-*α* resulted in the spontaneous development of a chronic erosive arthritis in 100% of the animals, with lesions similar to RA [27].

Before considering the impact of infection with helminths on joint inflammation, we must first briefly outline the immune response to infection with these metazoan parasites, as this is important to subsequent analyses of how “bystander' diseases can be modified by the parasite or the host response to the infection [1].

## 3. Mammalian Immune Response to Helminth Parasites

Although the precise nature of the host immune response varies considerably between species, a Th2 phenotype is a conserved response to helminth infection in mice and humans and is marked by the production of significant amounts of interleukins (IL)-4, -5, -9, -10, and -13 [1, 28], and the more recently identified IL-21 and IL-33. Mucosal mast cell hyperplasia, eosinophilia, IgE production, and increased expression of goblet cells are immunologic responses characteristic of infection with parasitic helminths [29]. Basically, there exist two potential mechanisms by which parasites can affect host immunity in a manner that would modify concomitant disease. First, products secreted from the parasite could have immunomodulatory effects; that is, they exert the capacity to directly modulate host immune functions. Second, the immune response generated to combat the infection could counteract the immunopathological reactions that drive autoimmune diseases. A widely recognized feature of helper T cells is that, in the presence of Th2 effector responses, Th1 responses are suppressed and vice versa. Thus, Th2-type reactions evoked in response to helminth infection would in theory have the ability to suppress proinflammatory Th1 responses that generate immunopathology. Models that incorporate infection with helminth parasites in conjunction with the expression of autoimmune diseases are proving to be extremely useful in elucidating immuno-regulatory pathways. Indeed, a picture is emerging in which infection with helminth parasites leads to a general dampening of host immunity or the creation of an immuno-regulatory environment (dominated by IL-10, transforming-growth factor (TGF-*β*), regulatory T cells, and alternatively activated macrophages) that can explain the seemingly paradoxical observation that the “helminth therapy” may reduce Th2-dominated conditions such as allergy and atopy [1, 2]. The goal of research with these animal models is that characterization of the host-parasite relationship will lead to the identification of therapeutic agents to control proinflammatory events.

### 3.1. Parasite-Derived Immunomodulatory Molecules

Virtually, all helminths studied to date express one or more molecules that can modify the activity of their host's immune response; however, the majority of these are poorly characterized [30, 31]. A notable exception is ES-62, a glycoprotein derived from *Acanthocheilonema vitea*. The numerous immuno-regulatory capacities of ES-62 are attributed to the presence of phosphorylcholine [32]. Features of ES-62-induced immune modulation include altered B-cell and cytokine proliferation, a shift towards an anti-inflammatory phenotype of antigen presenting cells, and reduced mast cell degranulation. Mechanistically, the action of ES-62 is in part attributed to its ability to downregulate protein kinase C, a signalling enzyme that plays a role in B cell proliferation and mast cell degranulation [33, 34]. In murine models, ES-62 induces a cytokine and antibody shift characterized by increased levels of IL-10, reduced levels of proinflammatory cytokines (IL-12, TNF*α*), and the production of IgG1 molecules [34]. Thus, ES-62 does not simply function as an immunosuppressant; rather it induces active anti-inflammatory responses via Th2-driven events. 

Administration of ES-62 can protect mice from collagen-induced arthritis through its inhibition of the production and activity of proinflammatory cytokines (e.g., IL-6, TNF*α*, and IFN*γ*) and collagen-specific antibodies and evoking the increased synthesis of IL-10 [35]. In terms of the relief of the symptoms of arthritis, ES-62 treatment resulted in reduced progression of knee swelling with the involvement of fewer joints, and synovial hyperplasia, cellular infiltration, and bone erosion were suppressed. In the presence of ES-62, cultures from human RA synovial fluid and membranes expressed reduced levels of TNF*α*, IL-6 [35], and IFN*γ* [36], while peripheral blood mononuclear cells from patients with RA exhibited low IFN*γ* secretion following ES-62 challenge [36].

Despite the myriad of partially purified helminth-derived molecules that can suppress or redirect mammalian immune responses, the promise of helminths as a source of anti-inflammatory or immunomodulatory drugs has yet to materialize [30, 31]. One reason for this has been the inability to isolate pure antigens/molecules from small amounts of parasite tissue; however, we contend that this should not deter the search for these molecules. Technological advances in recent years (e.g., mass spectrometry) have enhanced our ability to isolate, characterize, and purify molecules from miniscule amounts of starting material. Additionally, a wealth of simple bioassays is available to screen the potential immunomodulatory properties of these molecules.

## 4. Suppression of Joint Inflammation by Helminth Parasites

Numerous proof-of-principal studies have shown that the severity of concomitant disease in mice (e.g., colitis, airway hyper-reactivity, and experimental allergic encephalitis) can be reduced by prophylactic or therapeutic infection with parasitic helminths [2]. The mechanism of the reduction in disease has been, in a species- and model-specific manner, ascribed to the inhibition of Th1 cytokine production [37], induction of regulatory cytokines (e.g., IL-10 and TGF*β*) [38, 39], production of Foxp3^+^ regulatory T cells [40], and activity of alternatively activated macrophages [41]. (A smaller number of reports have presented the caveat that infection with helminth parasites can exaggerate other disease conditions [42].) To date we are aware of only five publications that have assessed the effect of infection with helminth parasites on joint inflammation.

To our knowledge, the first report on reduced arthritic disease in helminth-infected rodents was the serendipitous observation that rats infected with the nematode *Syphacia obvelata* had less CFA-induced arthritis (as gauged by the number of joints involved and degree of swelling) [43]. The identification of the helminth was made following the observation that some of the CFA-treated rats had limited disease—that is, the investigators had not purposefully infected a cohort of animals; rather the infection was unintended.

Spontaneous arthritis develops in Murphy Roths Large (MRL)/lpr mice. Infection of these mice with either of the nematodes *Heligmosomoides polygyrus* or *Nippostrongylus brasiliensis* resulted in a slightly reduced incidence of arthritis and a reduction in synovial hyperplasia. Other significant features of arthritic disease, including pannus formation, cartilage erosion, and bone destruction, were not attenuated by infection with either helminth [44]. While attesting to the potential of infection with helminth parasites to reduce the severity of arthritic disease, this study revealed no specific anti-inflammatory mechanism of action.

Recently, Osada and colleagues demonstrated that infection with the trematode *Schistosoma mansoni* attenuated Col II-induced arthritis in mice [45]. In that study, naïve mice or those infected two weeks previously with *S. mansoni* were immunized with heterologous Col II. The infected animals developed less severe arthritis, as indicated by reduced limb involvement and swelling. While typical arthritic features such as synovial hyperplasia, inflammatory cell recruitment, and bone/cartilage destruction were present in uninfected Col II immunized mice, mice infected with *S. mansoni* were protected from arthritis as assessed by these measures [45]. Dose studies indicated that 4-5 pairs of *S. mansoni *were sufficient to provide an antiarthritic effect. By 8 and 12 weeks after immunization the levels of IgG_2a_, which are involved in the pathology of Col II-induced arthritis, were significantly lowered in *S. mansoni*-infected mice. Polyclonal stimulated splenic T cells from Col II-injected mice produced substantial amounts of IFN*γ*, IL-17A, and TNF*α*, and comparatively little IL-4 and IL-10. The reverse is true of T cells retrieved from Col II+*S. mansoni-*treated mice. In addition, quantitative PCR revealed upregulation of IL-1*β* and IL-6, and receptor activator of nuclear factor *κ*-B ligand (RANKL) in the paws of Col II arthritic mice that was not observed in the *S. mansoni*-infected mice. Analysis of IL-10 and TGF*β* mRNA revealed no differences between the groups, and somewhat, counterintuitively, there was a subtle increase in Foxp3 mRNA in paws from immunized mice that was not observed in Col II+*S. mansoni*-treated mice. Collectively, these data demonstrate the capacity of a prophylactic infection with *S. mansoni* to attenuate the severity of collagen-induced arthritic disease, which may be due to systemic suppression of proinflammatory cytokine production and enhanced synthesis of IL-10. Very similar results have been reported by He et al. [46], who showed inhibition of Col II-induced arthritis in mice infected seven weeks, but not two weeks, previously with *Schistosoma japonicum*.

Most recently it was found that infection with the rat tapeworm, *Hymenolepis diminuta* significantly attenuated CFA-induced arthritis [23]. In this model, CFA was injected directly into the knee of naïve Balb/c or C57/Bl6 mice. A subset of animals was infected 8 days previously with *H. diminuta*, a time-point at which immunological expulsion of the helminth is underway. CFA-treated mice displayed an increase in knee diameter of 20–40%, increased synovial blood flow, increased myeloperoxidase (MPO) activity (a measure of granulocyte infiltrate, typically neutrophils), and increased pain sensitivity; all of these signs and symptoms of joint inflammation were reduced in the parasitized mice [23]. Moreover, infection with *H. diminuta* was as effective as treatment with either the steroid dexamethasone or the NSAID indomethacin, at inhibiting the proarthritic effects of intra-articular injection of CFA. In addition, the antiarthritic effect of infection with *H. diminuta* was absent in mice lacking T and B cells or the *α*-chain of the IL-4 receptor, suggesting that the active adaptive immune response against the parasite had the bystander effect of antagonising the effects of CFA. Typical of the response to infection with helminth parasites, spleen cells from the infected mice produced significantly more IL-4 and IL-10 when challenged with the T cell-mitogen, concanavalin A, than splenocytes from CFA-only-treated or naïve mice [23].

A subsequent series of mechanistic studies employed (i) an *in vivo* neutralizing antibody against IL-10 and (ii) IL-10 KO mice and (iii) adoptive transfer of CD4^+^ T cells from *H. diminuta*-infected wild-type or IL-10 KO mice [23]. The outcome of these investigations supports the conclusions that the IL-10 component of the response to infection with *H. diminuta* is important for the inhibition of CFA-induced joint inflammation and that CD4^+^ T cells (which could be a source of IL-10) from infected mice can transfer protection against the proarthritic effects of CFA injection [23].

## 5. The Challenges Ahead

The concept of “helminth therapy” has generated significant interest among scientists, the public, and some patients for whom standard therapies have either limited effectiveness or have failed [47, 48]. While the study of helminth parasites in models of arthritis has lagged behind those of other diseases (e.g., IBD [49]), data from murine model systems indicate that infection with helminth parasites can alleviate the severity of joint inflammation. Further, studies in other models of arthritis are warranted. These should be complemented with clinical data and observations and ultimately carefully controlled double-blind clinical trails. At this juncture, beyond the suggestion of the value of small open-label studies with a defined helminth in a well-characterized group of patients with arthritis, it is, in our view, premature to advocate helminth therapy for joint inflammation. A major challenge in the field will be the identification and characterization of specific antigens from a given helminth that drives the protective response and those antigens/molecules that can serve as blueprints for drug design in the development of novel anti-inflammatories or analgesics for the treatment of an array of human maladies.

There are potential drawbacks to helminth therapy that need to be carefully assessed: (i) certain parasites (e.g., *S. mansoni*) are not viable candidates because of the disease they cause (this does not diminish their value in animal models for the elucidation of anti-inflammatory mechanisms and immunological principles), (ii) the spectre of iatrogenic infection, and (iii) the possibility that infection with specific parasites could be additive to an existing condition or promote the clinical manifestation of a disease or comorbidities. For instance, recent data suggest that helminth therapy could be contraindicated in treating a disease characterized by increased numbers of eosinophils [50]. Moreover, despite the many reports of infection with helminth parasites blocking disease in animal models, the exact mechanism of action is not well defined, and potential side effects [51], dosing regimens, the impact of other helminth or protozoan infections, and the immunological and nutritional status of the host are important issues that remain unexplored.

Assessments of the anti-inflammatory effect(s) of infection with helminth parasites have focused on immune factors: an obvious and important starting point [1, 2]. However, lipid-derived molecules, stromal (e.g., fibroblasts) and endocrine cells, and modulation of the normal microflora of the gut all have the potential to dampen disease propagating events. The role of these factors in helminth inhibition of concomitant inflammatory disease is unknown and should be the focus of future research endeavours.

## 6. Conclusions

While a few studies have shown that infection with helminth parasites can exaggerate existing disease, the consensus is that the generation of an immunoregulatory environment as a consequence of infection with parasitic helminths can reduce the severity of concomitant disease (an arrangement that would benefit parasites while they complete their lifecycle). The consideration of the impact of infection with helminth parasites on arthritic disease is limited, but the available data support the general concept of “helminth therapy”—or rather, that data obtained from models of arthritis and concomitant helminth infection have the potential to reveal novel approaches to treat arthritis. While a number of obvious caveats pertain to helminth therapy, there is no doubt that there is much to be learned from analyses of rodent-helminth model systems, and a “tolerant” view of “our wormy friends” may be warranted. This is an exciting area of translational research that will eventually generate novel data on anti-inflammatory, proresolution, or immunoregulatory mediators, cells and pathways that can be converted into new approaches to autoinflammatory and idiopathic human diseases. The notion of being able to prescribe a specific parasitic helminth to treat a disease in an individual whose immunological status has been carefully characterized could be a tantalizing aspect of future “personalised medicine.” However, we see this as a future possibility and not a future certainty and caution individuals to resist any temptation to infect themselves with a helminth parasite. Finally, we would emphasize that animal models need to be more rigorously employed to comprehensively define all immuneregulatory ramifications and potential side effects of infection with parasitic helminths in the context of other disease.

##  Conflict of Interests 

The authors have no financial or other conflicts to declare.

## Figures and Tables

**Table 1:** Summary of some of the most common rodent models of musclo-skeletal disease.

Principle	Model	Induction	Immunological features	Reference
Cartilage complex autoimmunity	Collagen-induced arthritis (CIA)	Immunization with homologous or heterologous type II collagen	(i) Increases in anticollagen AB (ii) Increases in complement, neutrophils, macrophages, B cells, and *α/β* TCR^+^/CD4^+^ T cells (iii) Increases in TNF*α*, TGF*β*_2_, IL-1, IL-6, IL-17 and IFN*γ*, and IL-12 (later phases)	[10–13]
	Proteoglycan-induced arthritis	IP injections in FCA at 0 and 1 weeks; IP injections in FIA at week 4 and 7	(i) Increases in CD4^+^ T cells and Th1-type cytokines, particularly IFN*γ*	[14]
	K/BxN serum transfer	IP injection of serum from KRNxNOD strain	(i) Increases in GPI antibodies, mast cells, neutrophils, TNF*α*, and IL-1	[15]

Infection	Mycoplasma infection	IV injection with *M. arthriticus *	(i) Increases in Th1 in susceptible mice Increases in Th2 in arthritis-resistant mice (ii) Suppression of T-lymphocyte proliferation and IL-2 Increases in IL-4 and IL-6	[16, 17]
	Staphylococcus infection	IV injection with *S. aureus *	(i) Increases in TNF*α*, TNF*β*, IL-1*β*, and IL-12	[18]

Bacterial fragment induced	Streptococcal cell wall (SCW)	IP/IA injection of SCW	(i) Increases in TNF*α*, TNF*β*, IL-1, IL-6, IFN*β*, and IFN*γ* (ii) Increases in CD4^*+*^ T cells in chronic, not acute arthritis	[19–21]
	Mycobacterium in FCA	IA injection	(i) Increases in circulating neutrophils, TNF*α*, IL-12p40, and IL-17	[22, 23]

Adjuvantinduced	Avridine	SC injection	(i) Activated macrophages, CD4^+^ T cells	[24]
	Pristane	SC (rats) or IP (mice) injection + booster 7 weeks later	(ii) Increases in TNF*α*, IL-1*β*, and IL-6	[25]
	Oil induced FIA	SC injection	(iii) Increases in macrophages, neutrophils, CD4^+^ T cells, B cells, TNF*α*, and IL-6	[24]

Genetic manipulation	TNF*α*	TNF transgenic strain; spontaneous	(i) Increases in TNF*α*, IL-6, and IL-1*β*	[21]

(AB: antibodies; CFA: Freund's complete adjuvant; FIA: Freund's incomplete adjuvant; GPI: glucose-6 phosphate isomerase; IA: intra-articular; IFN*γ*: interferon-gamma; IL: interleukin; IP: intraperitoneal; IV: intravenous; KXN: T-cell receptor transgene mouse strain; NOD: nonobese diabetic mouse strain; RA: rheumatoid arthritis; SC: subcutaneous; TGF*β*: transforming growth factor-beta; TNF*α*: tumour necrosis factor-alpha).
